# Versatile Gold Telluride Iodide Monolayer as a Potential Photocatalyst for Water Splitting

**DOI:** 10.3390/nano12111915

**Published:** 2022-06-03

**Authors:** Bingru Hai, Zhanying Yang, Bo Zhou, Lei Zhang, Aijun Du, Chunmei Zhang

**Affiliations:** 1 School of Physics, Northwest University, Xi’an 710069, China; hbr0313@sina.com (B.H.); zyyang@nwu.edu.cn (Z.Y.); zhoubo@nwu.edu.cn (B.Z.); 2 Shaanxi Key Laboratory for Theoretical Physics Frontiers, Xi’an 710069, China; 3 Institute of Modern Physics and Peng Huanwu Center for Fundamental Theory, Northwest University, Xi’an 710069, China; 4 School of Chemistry and Physics, Queensland University of Technology, Brisbane, QLD 4001, Australia; l203.zhang@hdr.qut.edu.au

**Keywords:** AuTeI, photocatalytic, water splitting, exciton

## Abstract

Two-dimensional materials promise great potential for photochemical water splitting due to the abundant active sites and large surface area, but few of the known materials meet the rigorous requirements. In this work, we systematically investigate structural, electronic, and optical properties of an experimentally unexplored 2D material, i.e., gold telluride iodide (AuTeI) monolayer using density functional theory and Bethe–Salpeter equation approaches. Bulk AuTeI is a layered material and was realized in experiments a few decades ago. However, its bandgap is relatively small for water splitting. We find the exfoliation of monolayer AuTeI from the bulk phase is highly favorable, and 2D AuTeI is dynamically stable. The bandgap of 2D AuTeI becomes larger due to the quantum confinement effect. Importantly, the edge positions of the conduction band minimum and valence band maximum of 2D AuTeI perfectly fit the water oxidation and reduction potentials, enabling it a promising photocatalyst for water splitting. Additionally, the exciton binding energy of 2D AuTeI is calculated to be 0.35 eV, suggesting efficient electron-hole separation. Our results highlight a new and experimentally accessible 2D material for potential application in photocatalytic water splitting.

## 1. Introduction

The continuous growth of global energy demands and serious environmental issues call for urgent developments of new sustainable energy resources. Hydrogen is regarded as an alternative clean fuel to replace traditional fossil fuels [1,2,3,4] due to the maximum energy density and environmental friendliness. Photocatalytic hydrogen production is a promising way to solve these problems. Since the first report of the splitting of water into H_2_ and O_2_ under visible light by Fujishima [5], tremendous research efforts have been made to search for more efficient photocatalysts in recent years. Up to now, many 3D bulk materials, such as TiO_2_ [6], BiVO_4_ [7], CdS [8], and Fe_2_O_3_ [9], have been identified as promising photocatalysts for water splitting. However, most of them suffer from low efficiency due to limited surface area and the lack of enough active sites. Hence, the search for new alternative photocatalytic materials is highly desirable [10,11].

Compared to 3D materials, 2D materials possessing a large surface area with abundant catalytic active sites and minimum carrier migration distance have attracted intensive research interest in water splitting [12]. So far, a number of 2D photocatalysts have been reported, such as III_2_–VI_3_ materials [13,14,15], PtSSe [15], PdSP [16,17], Pd_3_(PS_4_)_2_ [18], alkaline earth metal nitride [19], PdSeO_3_ [20], Penta-MS_2_ [21], SiCP_4_ [22], and C_3_S [23]. However, few of them can meet the rigorous criteria, such as optimal band gap for harvesting visible or infrared light, favorable band edges straddling the water redox potentials, structural stability, abundant active sites, and low recombination rate of photogenerated electron–hole pairs. Thus, the exploration of new photocatalytic active 2D materials is urgently needed, but currently remains a significant challenge.

Gold tellurium iodide (AuTeI) is a layered material that was experimentally synthesized over 40 years ago [24]. The bulk AuTeI crystallizes in a monoclinic configuration at a space group of P21/c. The bandgap in the bulk phase is reported to be 0.9–1.5eV [24,25] which is relatively small to guarantee the fitting of band positions to the water oxide and reduction potentials. Thus, 3D AuTeI cannot be used as a potential photocatalyst for water splitting. However, due to the quantum size effect, 2D AuTeI exfoliated from the 3D bulk phase might possess a suitable bandgap and band edge positions toward water splitting. So far, there is no exploration of 2D AuTeI both experimentally and theoretically. Therefore, it is of high significance and paramount importance to investigate structural, electronic, and optical properties to evaluate whether 2D AuTeI is a potential photocatalyst for hydrogen production.

The 2D AuTeI has not been synthesized and no experimental data are available now. We, for the first time, predict 2D AuTeI as potential photocatalyst for water splitting. In this work, we have carried out systematic studies on the geometry, stability, electronic structure, optical properties, and exciton effect of 2D AuTeI by using density functional theory (DFT) and Bethe–Salpeter equation (BSE) approaches. The AuTeI monolayer is found to be easily exfoliated from the bulk phase due to ultrasmall cleavage energy. The calculated phonon spectrum confirms its dynamic stability. In sharp contrast to 3D AuTeI, the edge positions of conduction band minimum (CBM) and valence band maximum (VBM) perfectly fit the water redox potentials due to the quantum size effect. Two-dimensional AuTeI also exhibits excellent optical absorption in the visible light region, which can be also substantially tuned by a mechanical strain. The electron-hole (exciton) binding energy is calculated to be 0.35 eV. Our results suggest that 2D AuTeI is a promising photocatalyst for water splitting [26,27].

## 2. Materials and Methods

All the calculations are carried out based on the DFT as implemented in the plane wave Vienna Ab initio Simulation Package (VASP) code (vasp5.4.4, contact:VASP Software GmbH Sensengasse 8/12 A-1090, Vienna, Austria) [28,29]. Generalized gradient approximation in the Perdew–Burke–Ernzerhof (GGA-PBE) exchange-correlation functional [30] is used for geometry optimization. A Gamma-centered 9 × 9 × 1 k-points grid is used for sampling the first Brillouin zone of 2D AuTeI. All the atoms are fully relaxed until the residual force and total energy are converged to 0.001 eV/Å and 10^−6^ eV, respectively. The accurate electronic band structure is obtained by using hybrid density functional theory based on the Heyd–Scuseria–Ernzerhof (HSE) functional [31,32]. The exfoliation energy (E_f_) of 2D AuTeI from the bulk counterpart is calculated based on the following equation:

(1) $$E_{f}=\frac{E_{2d}}{n_{2d}}-\frac{E_{3d}}{n_{3d}}$$

where E_2d_ and E_3d_ represent the total energies of monolayer and bulk AuTeI, respectively, and n_2d_ and n_3d_ denote the number of atoms in the unit cells of 2D monolayer and 3D bulk, respectively [33,34,35]. To better account for the long-range non-covalent bonding interactions, a zero-damped van der Waals (vdW) interaction is taken into consideration based on the Grimme scheme [36,37]. The plane-wave energy cut-off is set to be 300 eV. A vacuum layer of at least 15 Å is adopted to minimize the interaction between neighboring layers. The standard oxidation and reduction potentials of water splitting are employed [38], namely, O_2_/H_2_O = −5.67 eV and H^+^/H_2_ = −4.44 eV, as a reference for comparing to the predicted band edge positions of the 2D AuTeI. The excitonic properties and optical bandgap are obtained using Green’s function (GW method) and G0W0–Bethe–Salpeter equation (GW-BSE) approaches [39,40,41]. The exciton binding energy will be determined by comparing the quasi-particle band edge and the optical bandgap from the BSE calculation [42,43,44]. The phonon spectrum is computed by using the density functional perturbation theory (DFPT) approach as implemented in the Quantum-ESPRESSO package (v.7.0, www.quantum-espresso.org) [45]. The phonon spectrum reflects the collective displacements of atoms. Under the harmonic approximation theoretical framework, the frequency (ω) is proportional to $\sqrt{\frac{\beta }{m}}=\sqrt{\frac{1}{m}\frac{\partial^{2}E}{\partial x^{2}}}$, where ***β*** being the force constant. At equilibrium, the potential energy of the system should always increase with respect to any combinations of atomic displacements [46]. In this case, ω should be always positive. If $\frac{\partial^{2}E}{\partial x^{2}}<0$, which means that the system is in a relatively higher energy state, it will generate imaginary frequency in the phonon spectrum.

## 3. Results and Discussion

**Figure 1:**
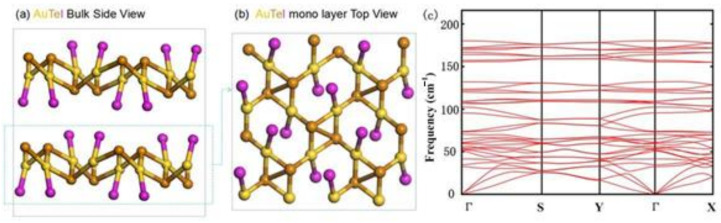
(**a**) The side view of the crystal structure of the AuTeI bulk in a 2 × 2 × 1 supercell; (**b**) top view of the 2D AuTeI monolayer. The yellow, brown, and purple balls represent Au, Te, and I atoms, respectively. (**c**) The calculated phonon spectrum for the AuTeI monolayer along the high-symmetry line in the first Brillouin zone.

The bulk AuTeI was experimentally fabricated a few decades ago and its crystal phase belongs to a monoclinic structure with the space group of P21/c (No.14) [24]. Figure 1a presents the primitive unit cell of bulk AuTeI containing 12 atoms. The bulk configuration comprises two monolayers stabilized by weak interlayer vdWs forces and features distorted square-planar coordination with bridging Te and terminal I atoms. The Au atom has an oxidation state of +3. The optimized geometrical structure of the 2D AuTeI monolayer is shown in Figure 1b. Detailed lattice parameters for bulk and monolayer AuTeI are obtained by the DFT calculations after the full relaxation of cells. As shown in Table 1, the lattice constants for bulk AuTeI are a = 7.55 Å, b = 7.83 Å, and c = 7.46 Å, which are in good agreement with the experimental values [24]. Compared to the bulk phase, the AuTeI monolayer has relatively smaller lattice parameters (a = 7.22 Å and b = 7.42 Å).

Having confirmed the equilibrium configurations of the 2D AuTeI, we then examine the possibility to exfoliate monolayer AuTeI from the bulk counterpart. First, the feasibility of mechanical exfoliation is investigated based on Equation (1) by calculating the exfoliation energy (E_f_), which reflects vdWs interactions strength in the bulk AuTeI phase. The exfoliation energy is defined as the energy cost of peeling the top layer from a surface of a bulk crystal [47]. The lower the formation energy is, the easier monolayer AuTeI can be exfoliated from the bulk phase. The E_f_ for the cleavage of AuTeI monolayer from the 3D bulk is calculated to be only 15 meV/atom, which is much smaller than that of experimentally successfully peeled graphene (~21 meV/Å^2^) and phosphorene (~22.7 meV/Å^2^) [44]. Such small cleavage energy demonstrates the high possibility of mechanically exfoliating the 2D AuTeI monolayer from its bulk phase. The dynamical stability of the AuTeI monolayer is further evaluated by calculating the phonon spectrum under the framework of DFPT. As shown in Figure 1c, there is no imaginary frequency in the phonon spectrum, indicating that single-layer AuTeI is dynamically stable.

**Figure 2:**
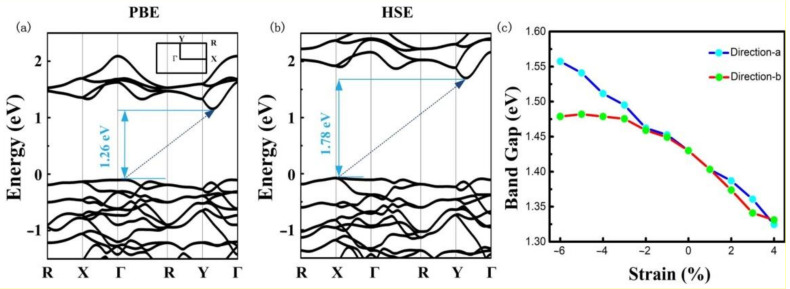
(**a**,**b**) The band structures of the 2D AuTeI calculated by PBE and HSE methods, respectively. The Fermi level is shifted at zero. The inset in (**a**) is the 2D Brillouin zone. (**c**) Band gap as a function of biaxial strain calculated with the PBE functional for the monolayer AuTeI.

**Figure 3:**
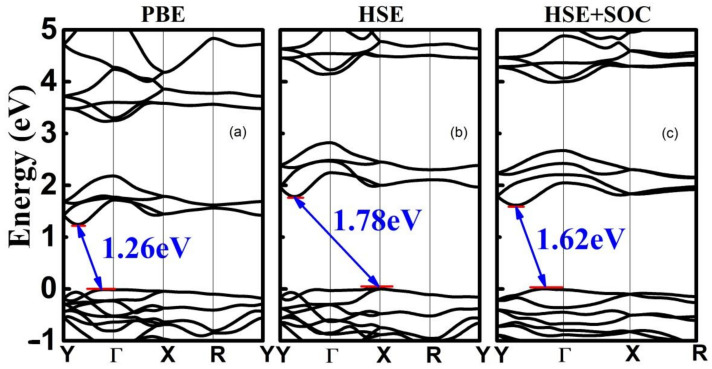
(**a**–**c**) The band structures of AuTeI monolayer calculated by PBE, HSE, and HSE + SOC methods, respectively. The Fermi level is set at zero.

In the following, we will systematically investigate the electronic properties of 2D AuTeI. Figure 2a presents the calculated band structure of 2D AuTeI at the PBE level. The AuTeI monolayer is found to be a semiconductor with an indirect gap of 1.26 eV, like monolayer MoS_2_, which has been exploited for photocatalytic applications due to its strong absorption in the solar spectrum region [48]. The valance band maximum (VBM) is situated along the wave vector Γ-R, while the conduction band minimum (CBM) locates at a point along the Y-Γ line. It is well known that the bandgap value is underestimated by the PBE functional. Then the HSE calculation is carried out to obtain a more accurate band structure as shown in Figure 2b. The bandgap of single-layer AuTeI is increased from 1.26 eV to 1.78 eV. It should be noted that the overall band dispersion under the HSE functional does not change at all compared to that by the PBE functional. Considering the spin-orbit coupling (SOC), the bandgap is 0.16 eV smaller than the pure HSE calculation shown in Figure 3.

**Figure 4:**
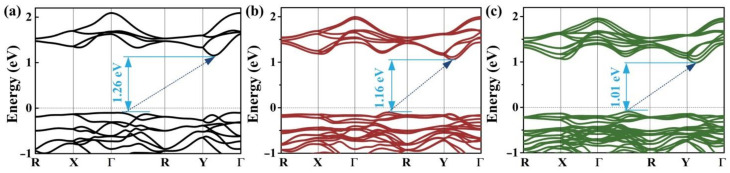
(**a**–**c**) Band structure for bilayer, trilayer, and bulk of AuTeI, respectively, calculated by the PBE functional method. The Fermi level is shifted at zero.

We also calculate the band structures for bilayer and trilayer AuTeI, as shown in Figure 4. They possess relatively small band gaps which are attributed to the well-known effect of quantum confinement that leads to the increasing bandgap from triple layer to monolayer. Additionally, the electronic properties of monolayer AuTeI are expected to be impacted by the external strain [49]. Figure 2c presents the change of bandgap in 2D AuTeI as a function of biaxial strain based on the calculations using the PBE functional. The bandgap of 2D AuTeI is very sensitive to the lattice strain. The bandgaps will shift downward with the increasing strain along with the directions of a and b lattice vectors. These suggest tunable electronic properties of monolayer AuTeI by a moderate mechanical strain, leading to potential applications in novel electronics or photovoltaic devices.

**Figure 5:**
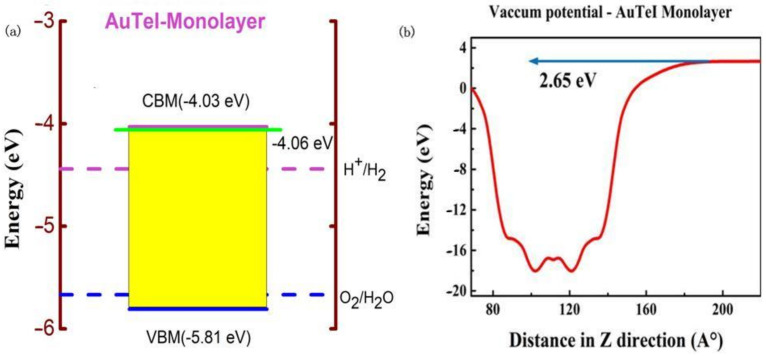
(**a**) Band positions of monolayer AuTeI calculated by the HSE06 functional compared to redox potentials of the water. The vacuum energy level is at 0 eV. The green line indicates the CBM position calculated by GW/BSE after considering exciton effect. (**b**) Electrostatic Vacuum potential for AuTeI monolayer.

The bandgap of AuTeI monolayer (1.78 eV) calculated by HSE functional indicates excellent optical absorption in the visible light region toward solar energy applications [50]. To further identify whether monolayer AuTeI is suitable for the use as efficient photocatalyst toward water splitting, we then accurately determine the band edge positions of VBM and CBM by aligning the HSE band positions concerning the vacuum energy level (Figure 5b) as shown in Figure 5a. The standard electrode potentials to produce H_2_ and O_2_ are also included. The band edge positions of the AuTeI monolayer stride the redox potentials of water, indicating excellent performance toward photocatalytic water splitting. By calculating energy differences between CBM and hydrogen reduction potential and between VBM and water oxidation potential, the reducing and oxidizing powers for the AuTeI monolayer are obtained to be 0.31 and 0.14 eV, which are large enough to trigger high-performance photocatalytic water splitting [51]. The CBM band edge position would, therefore, be shifted when the electron–hole binding energy is introduced and the revised CBM is marked in Figure 5a (green line). It is evident from the Figure 5a that after considering the effect of e–h binding energy on the shifting of CBM, the new CBM position is close to the CBM position calculated by HSE method and still facilitates the desired hydrogen evaluation. 

**Figure 6:**
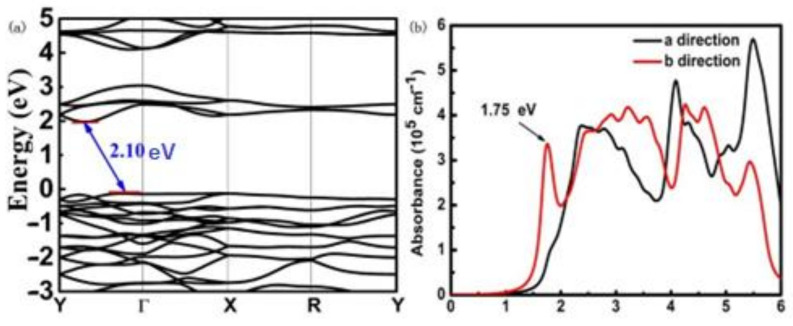
(**a**) GW-band structure for AuTeI monolayer with an indirect bandgap of 2.10 eV, and (**b**) BSE-optical absorption spectrum with an optical band gap of 1.75 eV.

The excitons, known as the electro-hole pairs or quasiparticles (QPs), that are generated during photoexcitation, generally have a strong electron-hole binding due to mutual electrostatic force, which is one of the critical indicators to control the charge separation in solar energy application. Here we use the GW approximation to accurately calculate band structure for 2D AuTeI as shown in Figure 6a. The optical absorption spectrum is then obtained by using a high-level BSE approach as presented in Figure 6b. The exciton energy of the AuTeI monolayer will be determined by evaluating the energy difference between the QP energy and the optical band gaps. It can be seen clearly from Figure 6a,b, the QP and optical band gaps of the AuTeI monolayer are around 2.10 eV and 1.75 eV, respectively. Therefore, the exciton binding energy [52,53,54] is derived to be 0.35 eV. It is important to note that semiconductors with exciton energies in the range of a few hundred milli-electron-volts are supposed to play a critical role in the charge separation in the water-splitting process. At the same time, due to the indirect bandgap of the 2D AuTeI, the emission of excited electrons to the valence band must be assisted by the phonon generation, thus prolonging the lifetime of electron–hole pairs and improving the photocatalytic efficiency [55]. Additionally, as shown in Figure 6b, single layer AuTeI possesses an excellent visible light response. This suggests that the AuTeI monolayer is capable of harvesting solar light. Along with the perfect fitting of band positions with water oxidation and reduction potential, monolayer AuTeI could be a versatile photocatalyst for water splitting.

## 4. Conclusions

In summary, we, for the first time, predict a new photochemically active and experimentally accessible 2D material, i.e., AuTeI monolayer for water splitting using DFT and BSE approaches. Monolayer AuTeI is found to be dynamically stable and could be easily exfoliated from the bulk counterpart due to smaller cleavage energy than that of graphene/phosphorene. Although bulk AuTeI is not suitable for water splitting, monolayer AuTeI possesses perfect positions of the CBM and VBM that fit the water oxidation and reduction potentials due to the well-known quantum size effect. Additionally, the exciton binding energy of 2D AuTeI is calculated to be only 0.35 eV, suggesting an efficient electron-hole separation. Our results highlight a new 2D material for the experiment to realize with great potential for photocatalytic water splitting.

## Figures and Tables

**Table 1:** The calculated and experimental values of lattice parameters for bulk and monolayer AuTeI.

	Bulk (Cal.)	Bulk (Exp.)	Monolayer (Cal.)
a (Å)	7.55	7.31	7.22
b (Å)	7.85	7.62	7.42
c (Å)	7.46	7.26	

## Data Availability

The data presented in this study are available in the articles.
